# Meta-analysis of the effects of physical activity on ocular biometrics in children and adolescents

**DOI:** 10.3389/fpubh.2025.1615033

**Published:** 2025-06-11

**Authors:** Pengfei Nie, Tong Feng

**Affiliations:** ^1^Nantong Institute of Technology, Nantong, China; ^2^Department of Physical Education, Kunsan National University, Gunsan-si, Republic of Korea; ^3^Beijing Sport University, Beijing, China

**Keywords:** physical activity, eye health, children, adolescents, meta-analysis, ocular biometrics, vision

## Abstract

**Introduction:**

Physical activity is recognized as an effective strategy for preventing myopia and cardiovascular diseases in children and adolescents; however, its impact on ocular biological parameters in this population remains poorly understood. This study systematically evaluates the effects of physical activity on ocular parameters in children and adolescents, aiming to provide a theoretical foundation for myopia and cardiovascular disease prevention.

**Methods:**

The study was registered on PROSPERO (CRD4202454649). A comprehensive search of electronic databases—Web of Science, Embase, Cochrane Library, and PubMed—was conducted from their inception through April 2025. Two reviewers independently performed literature screening, data extraction, and risk-of-bias assessments using predefined inclusion/exclusion criteria. Methodological quality was evaluated using the PEDro and NOS scales, and outcomes were analyzed via network meta-analysis in RevMan 5.3.

**Results:**

Fourteen studies [8 randomized controlled trials (RCTs), 3 cross-sectional studies, 2 non-randomized controlled trials (NRCTs), and 1 cohort study] involving 12,548 participants aged 6–26 years were included. Meta-analysis revealed that physical activity significantly improved KVA (MD = 0.23, 95% CI = 0.18–0.29, *p* < 0.001), enhanced UDVA (MD = 0.2, 95% CI = 0.08–0.32, *p* < 0.001), delayed axial length (AL) progression (MD = 0.2, 95% CI = 0.08–0.32, *p* < 0.001), suppressed spherical equivalent refraction (SER) progression (MD = 0.2, 95% CI = 0.08–0.32, p < 0.001), reduced central retinal venular equivalent (CRVE) (MD = −2.50, 95% CI = −4.86 to −0.13, *p* = 0.04), and narrowed central retinal arteriolar equivalent (CRAE) (MD = −2.50, 95% CI = −4.86 to −0.13, p = 0.04). Physical activity demonstrably enhances dynamic and uncorrected distance vision, attenuates SER progression, and positively influences retinal vascular parameters.

**Conclusion:**

Regular physical activity effectively slows myopia development and progression in children and adolescents while contributing to the prevention of cardiovascular diseases.

**Systematic review registration:**

https://www.crd.york.ac.uk/PROSPERO/view/CRD42024546449.

## 1 Introduction

Eye health represents a global public health priority, influencing not only individual well-being but also sustainable development and social equity (1). By 2050, the global myopic population is projected to reach 4.758 billion (49.8% of the global population), with 938 million individuals (9.8%) expected to have high myopia-a significant increase in prevalence from 2000 to 2050 (2). Recent epidemiological studies highlight pronounced geographic disparities in childhood myopia prevalence, with notably elevated rates in East Asian populations (3). While Asia’s prevalence surpasses that of Africa, Europe, and the Americas, regions such as Western Europe, Northern Europe, and the United States have also reported rising myopia rates and a declining age of onset (4, 5). Myopia progression in childhood typically continues through adolescence, exacerbating risks of ocular pathologies such as glaucoma, cataracts, and retinal detachment, which can impair educational and economic opportunities and increase healthcare burdens (6, 7).

Key ocular biometric parameters in pediatric populations include kinetic visual acuity (KVA), uncorrected distance visual acuity (UDVA), axial length (AL), central retinal arteriolar equivalent (CRAE), central retinal venular equivalent (CRVE), arteriolar-to-venular ratio (AVR), and spherical equivalent refraction (SER). KVA and UDVA assess ciliary muscle function (56, 62), with KVA offering predictive value for UDVA (8, 61). AL is a critical determinant of refractive status (9), while CRAE and CRVE serve as early markers of microvascular dysfunction (10). Reduced AVR is associated with hypertension-related morbidity and cardiovascular mortality (11). Myopia is commonly quantified using spherical equivalent refraction (SER), with a cutoff of ≤ − 0.50 diopters (D) indicating myopia (12, 13).

Physical activity —defined as skeletal muscle-driven movement—protects visual function through mechanisms such as improved ciliary muscle accommodation (57) and transient reductions in AL (67). Despite World Health Organization (WHO) recommendations of ≥60 min of daily physical activity for youth, 80% globally fail to meet this threshold (14, 60). Exercise modalities like cycling, walking, and jogging reduce intraocular pressure (IOP) (15–17), while moderate-intensity physical activity attenuates IOP and AL progression (67). Cross-sectional studies further link aerobic training to increased CRAE and reduced CRVE, suggesting enhanced retinal microvascular health (18, 19).

As a non-pharmacological intervention, physical activity enhances neurological and cardiovascular function, underscoring its potential for myopia (64) and cardiovascular disease prevention. A prior systematic review (20) postulated that physical activity exerts beneficial effects on central retinal arteriolar and venular equivalents (CRAE, CRVE). However, the included studies exhibited notable limitations, including insufficient empirical data on other ocular biometric parameters (KVA, UDVA, AL, SER) as well as a lack of systematic exploration and comprehensive evaluation of the associations between these parameters and physical activity. To address this critical gap, the present investigation employs a rigorous systematic review and meta-analysis to synthesize empirical evidence on the multifaceted impacts of physical activity across six ocular biometric markers: KVA, UDVA, AL, SER, CRAE, and CRVE. By elucidating the multidimensional mechanisms through which physical activity contributes to myopia prevention and cardiovascular health promotion in children and adolescents, this work provides a more robust evidentiary foundation and lays the theoretical groundwork for the formulation of public health intervention strategies.

## 2 Materials and methods

This review was conducted and reported in accordance with the PRISMA (Preferred Reporting Items for Systematic Reviews and Meta-Analyses) guidelines (21) and registered prospectively with PROSPERO (CRD42024546449).

### 2.1 Search strategy

Two investigators independently searched Web of Science, PubMed, Embase, and the Cochrane Library from database inception through April 22, 2025, covering all available literature. Search terms included Boolean combinations of keywords such as (“sport” OR “exercise” OR “physical activity”) AND (“vision” OR “ocular” OR “myopia” OR “nearsightedness”) (Refer to the Supplementary material). To establish the pertinent terminology, keywords were curated from high-caliber empirical studies and comprehensive literature reviews evaluating the effects of physical activity on ocular biophysical parameters (20, 22, 23). Manual screening of titles, abstracts, and full texts identified 51 studies for inclusion.

### 2.2 Eligibility and study selection

The PICOS (Population, Intervention, Comparison, Outcomes, Study design) framework guided inclusion criteria: (1) Participants aged around 6 to 18 years old; (2) Physical activity or outdoor interventions; (3) Activity-based interventions vs. non-intervention controls; (4) KVA, UDVA, AL, SER, CRAE, or CRVE; (5) Study design: Observational, cohort, randomized, or non-randomized trials. Exclusions included non-English articles, reviews, animal studies, adult/disease-specific cohorts, and studies lacking analyzable data.

### 2.3 Data screening and extraction

Two reviewers independently screened literature and extracted data following predefined protocols. Discrepancies were resolved through consultation with a third reviewer. Extracted data included study characteristics (author, year, design), participant demographics (age, sample size), intervention details (duration, outcomes, instruments), and outcome metrics (means, SDs, SEs).

### 2.4 Assessment of study quality

RCTs and non-RCTs were evaluated using the 11-item PEDro scale (54, 58, 63) (scores 0–10), with scores ≥6 indicating high quality, 4–5 moderate, and ≤3 low quality (24). Cohort and cross-sectional studies were assessed via the Newcastle-Ottawa Scale (NOS) (59), which assigns up to 9 stars across selection (4 stars), comparability (2 stars), and outcome (3 stars) domains. Two reviewers independently conducted assessments, with disagreements resolved by a third investigator.

### 2.5 Data analysis

Analyses were conducted using RevMan 5.3. Mean differences (MD) with 95% confidence intervals (CI) were calculated. Heterogeneity was assessed via the Q test and I^2^ statistic (I^2^ < 25% = low; I^2^ = 50% = moderate). Fixed- or random-effects models were applied based on I^2^ thresholds (55). Sensitivity analyses excluded individual studies to assess robustness. Publication bias was not evaluated due to limited study counts (<10 per outcome) (25).

## 3 Results

### 3.1 Flowchart and findings of literature selection

**Figure 1:**
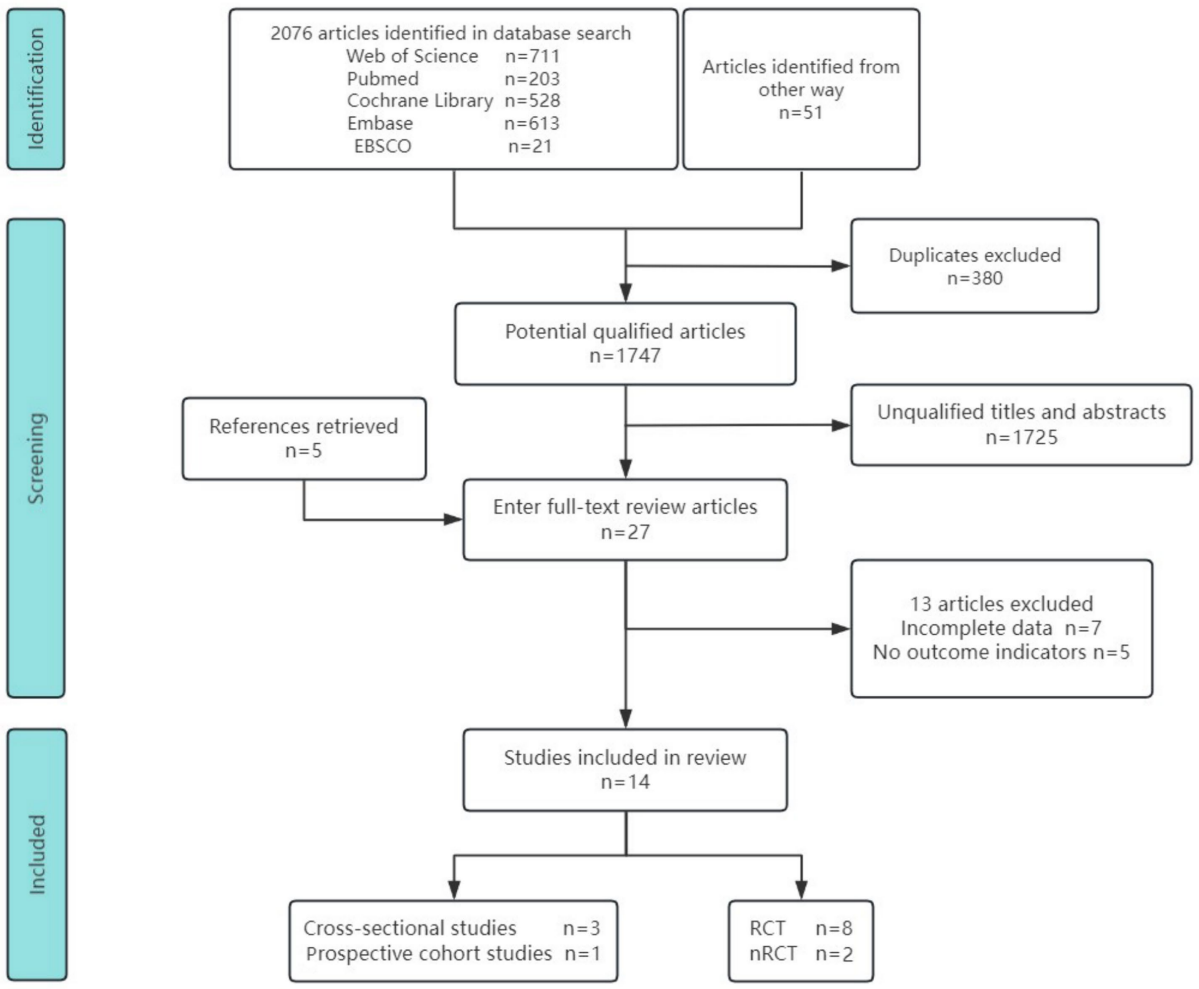
Screening of studies for inclusion.

We identified 2,076 records from five databases and 51 additional sources. After removing 380 duplicates using EndNote X9, 1,747 articles remained for screening. Initial title and abstract screening identified 28 potentially eligible studies, of which 14 were retained following full-text review (10, 13, 18, 22, 23, 26–34). The included studies comprised 3 cross-sectional analyses, 1 prospective cohort study, 8 randomized controlled trials (RCTs), and 2 non-randomized controlled trials (NRCTs). The studies assessed the following endpoints: kinetic visual acuity (KVA), uncorrected distance visual acuity (UDVA), axial length (AL), spherical equivalent refraction (SER), central retinal arteriolar equivalent (CRAE), and central retinal venular equivalent (CRVE). Figure 1 outlines the search strategy and study selection process.

### 3.2 Characteristics of included studies

This network meta-analysis included 14 studies published between 2008 and 2023. The pooled sample comprised 12,548 children and adolescents. Study characteristics are summarized in Tables 1, 2. By region, seven studies were conducted in mainland China, two in Taiwan, two in Australia, and one each in Germany, Switzerland, and Denmark. Interventions included four outdoor activities (e.g., rope skipping, badminton). Other interventions comprised sports-based games, physical education classes, kinetic visual sports (with cognitive tasks), home-based exercises, and lifestyle education programmes. The interventions lasted from 2 weeks to 3 years, were delivered 3–7 times per week, and consisted of 30-60 min sessions. Furthermore, physical activity influenced multiple ocular biomarkers, including kinetic visual acuity (KVA), uncorrected distance visual acuity (UDVA), axial length (AL), spherical equivalent refraction (SER), arteriole-venule ratio (AVR), central retinal arteriolar equivalent (CRAE), central retinal venular equivalent (CRVE), refractive error (RE), intraocular pressure (IOP), and eyestrain. Of these biomarkers, seven studies measured axial length, five examined spherical equivalent refraction, three investigated the impact on kinetic and uncorrected distance visual acuity, and three assessed effects on central retinal arteriolar and venular equivalents. Additionally, two studies assessed refractive error and arteriole-venule ratio, and one evaluated changes in intraocular pressure and eyestrain following physical activity. By design, eight were randomized controlled trials, two were non-randomized controlled trials, three were cross-sectional studies, and one was a prospective cohort study.

**Table 1:** Descriptive characteristics of included studies.

Study	Country/region	Research desgin	Participants characteristics			Intervention measures				Outcome indicators
			Gender	Age range	Sample size (T/C)	Intervention (T/C)	Cycle (week)	Frequency (Time/week)	Duration (minutes)	
Zhou (23)	China, Suzhou	RCT	NA	10–11	T = 115, C = 38	Ciliary muscle training combined with physical education classes/Regular physical education classes	32	3	40	UDVA, KVA, AL
Wu et al. (53)	China, Taiwan	RCT	M-52.15%	6–7	T = 267, C = 426	11 h of outdoor activity per week, with an additional 150 min of exercise time each week/ROCT711 intervention was not conducted	52	NA	NA	The changes in SER and AL, as well as the variations in outdoor light intensity and duration
Yin et al. (33)	China, Suzhou	RCT	NA	9–10	T = 43, C = 43	Physical activities (basketball/soccer) combined with ciliary muscle adjustment training/Regular physical activities	16	3	40	UDVA, KVA
Yin et al. (22)	China, Suzhou	RCT	M-59%	10–11	T = 127, C = 33	Ciliary muscle training combined with open-skill exercises and closed-skill exercises/Regular physical activities	16	3	40	UDVA, KVA
Zheng et al. (34)	China, Guangdong	RCT	M-52.3%	12–13	T = 467, C = 429	Home quarantine physical activities and REAP live broadcasts/Health information course (home exercise)	2	7	15*4	Eye strain
He et al. (28)	China, Guangzhou	RCT	M-54%	6–7	T = 902, C = 913	Outdoor activity course/Continue the previous activity mode	156	NA	NA	The changes in myopia rate, spherical SER, and AL
Jin et al. (30)	China, Shenyang	RCT	M-51%	6–11	T = 214, C = 177	Outdoor activities (skipping rope and badminton)/No intervention measures	52	5	40	The incidence of new myopia RE, AL, IOP
Siegrist (10)	Germany	RCT	M-60%	10–11	T = 241, C = 191	Lifestyle course, with increased physical activities both on and off campus/JuvenTUM 3	78	NA	NA	AVR, CRVE, CRAE
Guo et al. (27)	China, Beijing	nRCT	M-49%	6–7	T = 157, C = 216	Jogging/No intervention measures	52	5	30	AL, RE
Wu et al. (32)	China, Taiwan	nRCT	M-51%	7–11	T = 333, C = 238	Outdoor activity (ROC)/Routine activities	104	NA	NA	SER, AL

T, experimental group; C, control group; UDVA, uncorrected distance visual acuity; KVA, kinetic visual acuity; AL (mm), axial length; SER, spherical equivalent refraction; RE, refractive error; IOP (mmHg), Intraocular pressure; AVR, arteriolar-to-venular ratio; CRVE (μm), central retinal venular equivalents; CRAE (μm), central retinal arteriolar equivalents; NA, not applicable.

**Table 2:** Descriptive characteristics of included studies.

Study	Country/region	Research desgin	Participants characteristics			Testing method	Outcome indicators
			Gender	Age range/mean ± sd	Sample size		
Jacobsen (29)	Denmark	Prospective cohort study	M-39%	23.1 ± 3.3	Total = 143	Power bike	SER, AL
Rose (13)	Australia	Cross-sectional study	NA	6 and 12	6 year = 1735	NA	SER
					12 year = 2,353		
Gopinath (26)	Australia	Cross-sectional study	M-50.7%	6–7	Total = 1,492	NA	CRAE、CRVE
Imhof (18)	Switzerland	Cross-sectional study	M-53%	7.3 ± 0.4	Total = 1,255	20-m Shuttle run	AVR, CRAE, CRVE

AL (mm), axial length; SER, spherical equivalent refraction; AVR, arteriolar-to-venular ratio; CRVE (μm), central retinal venular equivalents; CRAE (μm), central retinal arteriolar equivalents; NA, not applicable.

### 3.3 Assessment of methodological quality

Quality appraisal was conducted using the Physiotherapy Evidence Database (PEDro) and the Newcastle-Ottawa Scale (NOS); the PEDro scores are shown in Table 3 and the NOS scores in Table 4. All studies scored at least six points on the PEDro scale, with randomized controlled trials averaging seven points, indicating robust methodological quality. Prospective cohort and cross-sectional studies achieved NOS scores ranging from six to nine stars; the NOS classification defines 0–3 stars as low quality, 4–6 as moderate, and 7–9 as high quality (65). The mean NOS score was eight stars, denoting excellent study quality.

**Table 3:** Summary of methodological quality of the included studies according to the PEDro scale (*n* = 11).

Study	Eligibility criteria	Random allocation	Concealed allocation	Baseline comparability	Blinded subjects	Blinded therapist	Blinded assessor	Sufficient follow-up sample size	Intention-to-treat analysis	Intergroup comparison	Estimation and variability	Total score/11
Zhou (23)	Y	Y	N	Y	N	N	N	Y	Y	Y	Y	7
Wu et al. (53)	Y	Y	Y	Y	N	N	Y	Y	Y	Y	Y	9
Yin et al. (33)	Y	Y	N	Y	N	N	N	Y	Y	Y	Y	7
Yin et al. (22)	Y	Y	N	Y	N	N	N	Y	Y	Y	Y	7
Zheng et al. (34)	Y	Y	Y	Y	N	N	Y	Y	Y	Y	Y	9
He et al. (28)	Y	Y	Y	Y	N	N	N	Y	Y	Y	Y	8
Jin et al. (30)	Y	Y	N	Y	N	N	N	Y	Y	Y	Y	7
Siegrist et al. (10)	Y	Y	Y	Y	N	N	N	Y	Y	Y	Y	8
Guo et al. (27)	Y	N	N	Y	N	N	N	Y	Y	Y	Y	6
Wu et al. (32)	Y	N	N	Y	N	N	N	Y	Y	Y	Y	6

Each item is scored as 1 point(Y) for compliance and 0 points (N) for non-compliance; the highest score is 9 points it is not possible to blind subjects and therapists in intervention studies.

**Table 4:** The methodological quality of cohort studies and cross-sectional studies included in the research is summarized according to the NOS (Newcastle-Ottawa Scale) assessment scale (*n* = 4).

Study	Selection				Outcom			Comparability	Total score (9)
	Representativeness of the exposed cohort (★)	Selection of the non exposed cohort (★)	Ascertainment of exposure (★)	Demonstration that outcome of interest was not present at start of study (★)	Assessment of outcome (★)	Was follow-up long enough for outcomes to occur (★)	Adequacy of follow up of cohorts (★)	Comparability of cohorts on the basis of the design or analysis (2★)	
Rose (13)	★	★	★		★	★	★	★★	8
Gopinath (26)	★	★	★	★	★	★	★	★★	9
Imhof (18)		★	★	★	★			★★	6
Jacobsen (29)	★	★	★	★	★	★	★	★★	9

In the two main categories of selection and outcomes, each subcategory can earn a maximum of 1★, with comparability earning a maximum of 2★. The highest possible score is 9★.

### 3.4 Results of the meta-analysis

#### 3.4.1 Impact of physical activity on KVA

**Figure 2:**
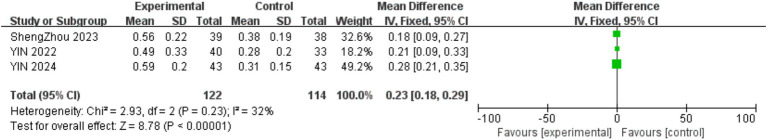
Impact of physical activity on KVA.

Three studies examined the impact of physical activity interventions on kinetic visual acuity (KVA) among 399 participants aged 9–11. As shown in Figure 2, physical activity interventions resulted in a significant improvement in KVA within the experimental cohort (MD = 0.23, 95% CI = 0.18–0.29, *p* < 0.001; I^2^ = 32%).

#### 3.4.2 Impact of physical activity on UDVA

**Figure 3:**
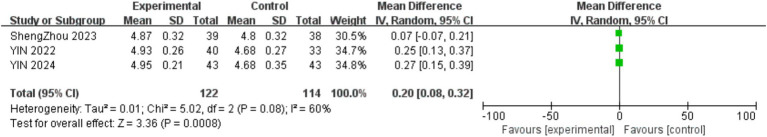
Impact of physical activity on UDVA.

**Figure 4:**
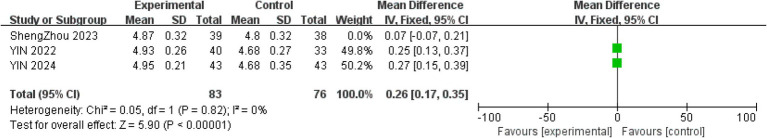
Sensitivity analysis.

Three trials evaluated uncorrected distance visual acuity (UDVA). Given that KVA partially predicts UDVA, physical activity interventions significantly improved UDVA (22). Figure 3 demonstrates that physical activity improved UDVA in the intervention group (MD = 0.2, 95% CI = 0.08–0.32, *p* < 0.001; I^2^ = 60%). To explore the sources of heterogeneity, sensitivity analysis was performed on the included studies, and each single study was excluded in turn (MD = 0.26, 95% CI = 0.17–0.35, I^2^ = 0%, *p* < 0.001; Figure 4).

#### 3.4.3 Impact of physical activity on AL

**Figure 5:**
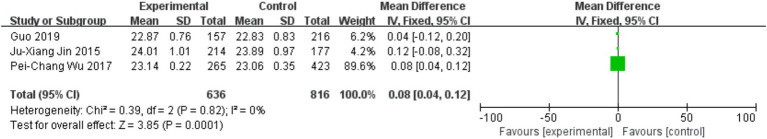
Impact of physical activity on AL.

Three trials assessed the effects of physical activity on axial length (AL) progression. Figure 5 indicates that sustained outdoor physical activity slowed AL elongation in youth (MD = 0.08, 95% CI = 0.04–0.12, *p* = 0.0001; I^2^ = 0%).

#### 3.4.4 Impact of physical activity on SER

**Figure 6:**
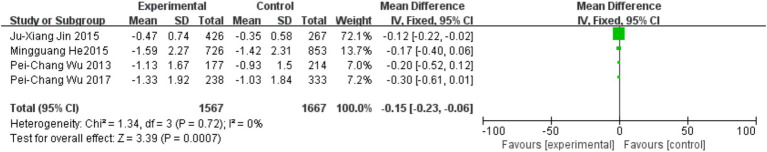
Impact of physical activity on SER.

Four trials investigated the effects of physical activity on spherical equivalent refraction (SER), all featuring intervention durations ≥1 year and focusing on outdoor exercise protocols. As illustrated in Figure 6, outdoor physical activity significantly slowed SER progression and reduced myopia incidence (MD = −0.15, 95% CI = −0.23 to −0.06, *p* = 0.0007; I^2^ = 0%).

#### 3.4.5 Impact of physical activity on CRVE and CRAE

**Figure 7:**
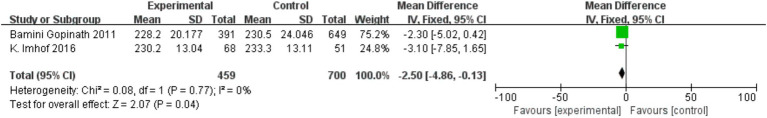
Impact of physical activity on CRVE.

**Figure 8:**
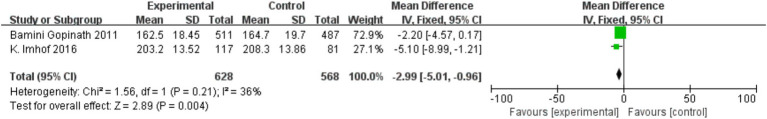
Impact of physical activity on CRAE.

Two cross-sectional studies investigated central retinal venular/arteriolar equivalents (CRVE/CRAE). Figure 7 shows physical activity was associated with reduced CRVE (MD = −2.50, 95% CI = −4.86 to −0.13, *p* = 0.04; I^2^ = 0%), while Figure 8 indicates increased CRAE diameters (MD = −2.99, 95% CI = −5.01 to −0.96, *p* = 0.004; I^2^ = −36%).

In a secondary analysis of 432 children aged 10–11, 18-month physical activity interventions resulted in significant arteriole-to-venule ratio (AVR) improvements in 83% of participants (mean increase: 0.88 ± 0.06 to 0.91 ± 0.06) and reduced CRVE dilation (*p* = 0.019) (10). Cross-sectional data associated increased outdoor time with higher mean SER in 12-year-olds (*p* = 0.003), though this trend was nonsignificant in 6-year-olds (*p* = 0.09) (13). physical activity levels were inversely associated with myopia severity (*p* = 0.015). Additionally, physical activity interventions reduced eyestrain severity in 896 adolescents aged 12–13 (MD = −0.15, 95% CI = −0.26 to −0.03, *p* = 0.02).

## 4 Discussion

Eye health constitutes a critical component of sustainable human development and universal health coverage, as visual acuity undergoes rapid postnatal development with age-specific patterns: UDVA exhibits a critical developmental window before age 6 and typically reaches full maturation by approximately 8 years of age (1). During ocular development and biometric changes throughout childhood and adolescence, this phase is particularly vulnerable; prolonged screen exposure, insufficient exercise, and reduced outdoor time may precipitate myopia onset, which progresses with age and accelerates markedly between 7 and 12 years (22, 35). Without timely intervention, myopia progression accumulates, increasing the likelihood of high-degree and pathological myopia, while visual impairment is itself associated with elevated cardiovascular and systemic health risks (36). We selected kinetic visual acuity (KVA), uncorrected distance visual acuity (UDVA), axial length (AL), spherical equivalent refraction (SER), CRAE, and CRVE as endpoints, as these metrics serve as comprehensive and objective biomarkers of ocular health and visual function. To address this, we conducted a systematic review and meta-analysis to evaluate how physical activity and outdoor interventions influence myopia prevention and retinal microvascular outcomes in children and adolescents.

Meta-analysis results demonstrate that structured physical activity interventions significantly improve both KVA and UDVA among young participants. These findings align with prior meta-analytic evidence (20) showing that exercise positively influences ocular biometrics in children and adolescents. Notably, our review highlights the integration of visual-cognitive tasks into school-based physical education-leveraging the structured, sustained framework of school PE-to effectively prevent and decelerate myopia progression in youth. Kinetic visual acuity, the ability to perceive details of objects moving backward and forward toward the eye, relies greatly on the ciliary muscle and is closely linked to its regulation (22, 66). The perception of moving objects is primarily dependent on the accommodative function of the ciliary muscle. Myopia development frequently coincides with impaired or dysregulated ciliary muscle accommodative function (23). Research indicates that specific accommodation exercises enhance ciliary muscle contractions and relaxations, thereby improving ocular perfusion and retinal image resolution. Following accommodation training, myopic patients exhibit enhanced positive relative accommodation (PRA), accommodative responsiveness, and precision-factors strongly associated with myopia improvement (37). Given that both targeted ciliary training and integrated visual-cognitive tasks during exercise incorporate distance-shifting mechanisms (67), specialized exercise regimens may substitute traditional accommodation training, enhancing ciliary muscle responsiveness, driving favorable biometric changes, and preserving vision (33). In pediatric and adolescent populations, exercise represents the most effective method for ciliary muscle conditioning (22). Following the integration of visual-cognitive components, both open- and closed-skill exercises significantly improve KVA and UDVA among youth, with dynamic acuity positively correlating with static acuity and KVA acting as a mediator-thereby facilitating UDVA improvement (38).

The analysis further revealed that outdoor activity interventions significantly inhibit axial length (AL) elongation, thus preventing myopia onset and progression in young populations. Research on the light-dopamine mechanism confirms the protective effects of outdoor light: intense illumination stimulates dopamine secretion, which suppresses AL growth, mitigating myopia onset and progression, whereas even low-intensity light in dim environments can inhibit myopia (39). Exposure to two hours of daily outdoor sunlight significantly reduces the risk of form-deprivation myopia and prevents myopia onset (40). Additionally, evidence indicates that light quality parameters (e.g., intensity, wavelength, distribution) differentially influence myopia development and progression. Modulation of illumination intensity, spectral composition, and distribution patterns represents an effective strategy to prevent and control myopia (41). In animal models, non-uniform illumination induces visual fatigue, leading to form deprivation, pronounced myopic shifts, and AL elongation, which promote myopia development (42). Evidence suggests that intermittent high-intensity illumination is more effective than continuous exposure in suppressing myopia progression (43). The study emphasizes the critical role of light regimens in myopia prevention, proposing that intermittent lighting enhances dopamine secretion and positively modulates ocular biometrics. Outdoor exercise naturally provides intermittent high-intensity illumination, corroborating its efficacy in protecting against myopia among youth.

Using CRAE and CRVE as endpoints, we found that physical activity interventions significantly improved retinal arterial and venular calibers. These parameters are recognized early biomarkers of microvascular dysfunction (10) and may evaluate cardiovascular risk in children and adolescents (44). Research indicates that altered retinal microvasculature (characterized by arteriolar narrowing and venular widening) is associated with cardiovascular disease risk (45). Nonetheless, the mode and intensity of physical activity interventions differentially influence CRAE and CRVE, with children who engage in greater outdoor activity and higher-intensity exercise exhibiting increased arteriolar caliber (26). The mechanism may involve exercise-induced enhancement of endothelial function (46), in which increased blood flow elevates shear stress, enhances nitric oxide synthesis, and improves endothelium-mediated vasodilation (47, 48). Thus, the association between outdoor activity and arteriolar enlargement suggests microvascular structural adaptation and modulation of endothelial dysfunction (49). In contrast, greater endurance capacity in children is associated with reduced venular caliber (18). Systemic inflammatory processes contribute to microvascular impairment in children, and the anti-inflammatory effects of exercise likely underpin the favorable relationship between endurance and venular narrowing (50). Among pediatric subjects, endurance activities may constrict retinal venules via anti-inflammatory pathways, distinct from endothelium-mediated arteriolar responses (51). In pediatric populations, increased CRVE is associated with elevated hypertension risk (52), suggesting that higher endurance capacity could mitigate cardiovascular risk and improve retinal microvascular integrity.

This study also has some limitations and shortcomings: (1) Although this study includes fourteen studies encompassing randomized controlled trials, non-randomized controlled trials, cross-sectional studies, and cohort studies, the small number of each type of study hinders the interpretation of heterogeneity and publication bias. (2) The significant differences in the forms and dosages of physical activity interventions (such as types of exercise, intensity, frequency, etc.) may affect the accuracy of the combined effect. (3) The physical activities included range from short-term live home exercises to medium to long-term outdoor running, jumping, and break activities, with durations varying from 2 weeks to 3 years; different types of physical activities may lead to content bias. (4) Nearly half of the included studies focus on mainland China and Taiwan, while other studies are distributed in Europe and Australia. There is still insufficient understanding of the relationship between vision and physical activity in non-East Asian or Western populations; cultural differences and variations in outdoor activity styles may affect the generalizability of the results. These shortcomings need further exploration and resolution in future research.

## 5 Conclusion

Our systematic review found that physical activity interventions positively influence six ocular biometric parameters: keratometric values (KVA), axial length (AL), uncorrected distance visual acuity (UDVA), spherical equivalent refraction (SER), central retinal arteriolar equivalent (CRAE), and central retinal venular equivalent (CRVE). Regular physical activity may help prevent the onset of myopia in children and adolescents, slow its progression, and contribute to favorable remodeling of the retinal microvasculature, thereby reducing future cardiovascular risk. Further research is needed to investigate the differential effects of various types of physical activity on specific ocular biometric indicators and to conduct long-term follow-up studies to confirm these positive impacts on visual health in pediatric populations.

## Data Availability

The original contributions presented in the study are included in the article/Supplementary material, further inquiries can be directed to the corresponding author/s.
