# Supplementary material for: Meta-analysis of the effects of physical activity on ocular biometrics in children and adolescents
Source: Front Public Health. 2025 Jun 11;13:1615033. doi: 10.3389/fpubh.2025.1615033 (PMC12187683; doi:10.3389/fpubh.2025.1615033)
Supplement: Supplementary file 1 [file Data_Sheet_1.ZIP › Search strategy.docx]

**web of science**

1: TS=(sports) and Preprint Citation Index (Exclude – Database) Results: 199714

2: TS=(Sport or Athletics or Athletic) and Preprint Citation Index (Exclude – Database) Results: 213058

3: #1 OR #2 and Preprint Citation Index (Exclude – Database) Results: 213058

4: TS=(excercise) and Preprint Citation Index (Exclude – Database) Results: 797

5: TS=(Exercises or Physical Activity or Activities, Physical or Activity, Physical or Physical Activities or Exercise, Physical or Exercises, Physical or Physical Exercise or Physical Exercises or Acute Exercise or Acute Exercises or Exercise, Acute or Exercises, Acute or Exercise, Isometric or Exercises, Isometric or Isometric Exercises or Isometric Exercise or Exercise, Aerobic or Aerobic Exercise or Aerobic Exercises or Exercises, Aerobic or Exercise Training or Exercise Trainings or Training, Exercise or Trainings, Exercise) and Preprint Citation Index (Exclude – Database) Results: 730471

6: #4 OR #5 and Preprint Citation Index (Exclude – Database) Results: 730668

7: #3 OR #6 and Preprint Citation Index (Exclude – Database) Results: 873322

8: TS=(Vision, Ocular) and Preprint Citation Index (Exclude – Database) Results: 18007

9: TS=(Vision or Ocular Vision or Light Signal Transduction, Visual or Visual Light Signal Transduction or Visual Transduction or Transduction, Visual or Visual Phototransduction or Phototransduction, Visual) and Preprint Citation Index (Exclude – Database) Results: 881258

10: #8 OR #9 and Preprint Citation Index (Exclude – Database) Results: 881258

11: TS=(Myopia) and Preprint Citation Index (Exclude – Database) Results: 13891

12: TS=(Myopias or Nearsightedness or Nearsightednesses) and Preprint Citation Index (Exclude – Database) Results: 421

13: #11 OR #12 and Preprint Citation Index (Exclude – Database) Results: 13969

14: #10 OR #13 and Preprint Citation Index (Exclude – Database) Results: 891495

15: #7 AND #14 and Preprint Citation Index (Exclude – Database) Results: 15996

16: TS=(randomized controlled trial or randomized or placebo or randomised or random) and Preprint Citation Index (Exclude – Database) Results: 1125999

17: #15 AND #16 and Preprint Citation Index (Exclude – Database) Results: 711

**embase**

| No. | Query |
| --- | --- |
| #13 | #11 AND #12 |
| #12 | 'randomized controlled trial':ab,ti OR randomized:ab,ti OR placebo:ab,ti OR randomised:ab,ti OR random:ab,ti |
| #11 | #5 AND #10 |
| #10 | #6 OR #7 OR #8 OR #9 |
| #9 | 'near sightedness':ab,ti OR nearsightedness:ab,ti OR 'short sightedness':ab,ti OR shortsightedness:ab,ti OR 'simple myopia':ab,ti OR myopia:ab,ti |
| #8 | 'myopia'/exp |
| #7 | 'capacity, visual':ab,ti OR 'central vision':ab,ti OR 'half vision':ab,ti OR 'ocular vision':ab,ti OR 'optic perception':ab,ti OR 'perception, optic':ab,ti OR 'perception, visual':ab,ti OR 'perceptual closure':ab,ti OR 'twilight vision':ab,ti OR 'vision, ocular':ab,ti OR 'visual capacity':ab,ti OR 'visual detection':ab,ti OR 'visual function':ab,ti OR 'visual perception':ab,ti OR 'visual performance':ab,ti OR 'visual process':ab,ti OR 'visual sensation':ab,ti OR vision:ab,ti |
| #6 | 'vision'/exp |
| #5 | #1 OR #2 OR #3 OR #4 |
| #4 | 'biometric exercise':ab,ti OR effort:ab,ti OR 'exercise capacity':ab,ti OR 'exercise performance':ab,ti OR 'exercise training':ab,ti OR exertion:ab,ti OR 'fitness training':ab,ti OR 'fitness workout':ab,ti OR 'physical conditioning, human':ab,ti OR 'physical effort':ab,ti OR 'physical exercise':ab,ti OR 'physical exertion':ab,ti OR 'physical work-out':ab,ti OR 'physical workout':ab,ti OR exercise:ab,ti |
| #3 | 'exercise'/exp |
| #2 | 'competitive gymnastics':ab,ti OR 'competitive sport':ab,ti OR sports:ab,ti OR sport:ab,ti |
| #1 | 'sport'/exp |

**Cochrane library**

#1 MeSH descriptor: [Sports] explode all trees

#2 (Sport):ti,ab,kw or (Athletics):ti,ab,kw or (Athletic):ti,ab,kw

#3 MeSH descriptor: [Exercise] explode all trees

#4 (Exercises):ti,ab,kw or (Physical Activity):ti,ab,kw or (Activities, Physical):ti,ab,kw or (Activity, Physical):ti,ab,kw or (Physical Activities):ti,ab,kw or (Exercise, Physical):ti,ab,kw or (Exercises, Physical):ti,ab,kw or (Physical Exercise):ti,ab,kw or (Physical Exercises):ti,ab,kw or (Acute Exercise):ti,ab,kw or (Acute Exercises):ti,ab,kw or (Exercise, Acute):ti,ab,kw or (Exercises, Acute):ti,ab,kw or (Exercise, Isometric):ti,ab,kw or (Exercises, Isometric):ti,ab,kw or (Isometric Exercises):ti,ab,kw or (Isometric Exercise):ti,ab,kw or (Exercise, Aerobic):ti,ab,kw or (Aerobic Exercise):ti,ab,kw or (Aerobic Exercises):ti,ab,kw or (Exercises, Aerobic):ti,ab,kw or (Exercise Training):ti,ab,kw or (Exercise Trainings):ti,ab,kw or (Training, Exercise):ti,ab,kw or (Trainings, Exercise):ti,ab,kw

#5 MeSH descriptor: [Vision, Ocular] explode all trees

#6 (Vision):ti,ab,kw or (Ocular Vision):ti,ab,kw or (Light Signal Transduction, Visual):ti,ab,kw or (Visual Light Signal Transduction):ti,ab,kw or (Visual Transduction):ti,ab,kw or (Transduction, Visual):ti,ab,kw or (Visual Phototransduction):ti,ab,kw or (Phototransduction, Visual):ti,ab,kw

#7 MeSH descriptor: [Myopia] explode all trees

#8 (Myopias):ti,ab,kw or (Nearsightedness):ti,ab,kw

#9 (randomized controlled trial ):ti,ab,kw or (randomized ):ti,ab,kw or (placebo ):ti,ab,kw or (randomised ):ti,ab,kw or (random):ti,ab,kw

#10 #1 or #2 or #3 or #4

#11 #5 or #6 or #7 or #8

#12 #9 and #10 and #11

**PubMed**

Search: (((("Exercise"[Mesh]) OR ((((((((((((((((((((((((Physical Activity[Title/Abstract]) OR (Activities, Physical[Title/Abstract])) OR (Activity, Physical[Title/Abstract])) OR (Physical Activities[Title/Abstract])) OR (Exercise, Physical[Title/Abstract])) OR (Exercises, Physical[Title/Abstract])) OR (Physical Exercise[Title/Abstract])) OR (Physical Exercises[Title/Abstract])) OR (Acute Exercise[Title/Abstract])) OR (Acute Exercises[Title/Abstract])) OR (Exercise, Acute[Title/Abstract])) OR (Exercises, Acute[Title/Abstract])) OR (Exercise, Isometric[Title/Abstract])) OR (Exercises, Isometric[Title/Abstract])) OR (Isometric Exercises[Title/Abstract])) OR (Isometric Exercise[Title/Abstract])) OR (Exercise, Aerobic[Title/Abstract])) OR (Aerobic Exercise[Title/Abstract])) OR (Aerobic Exercises[Title/Abstract])) OR (Exercises, Aerobic[Title/Abstract])) OR (Exercise Training[Title/Abstract])) OR (Exercise Trainings[Title/Abstract])) OR (Training, Exercise[Title/Abstract])) OR (Trainings, Exercise[Title/Abstract]))) OR (("Sports"[Mesh]) OR (((Sport[Title/Abstract]) OR (Athletics[Title/Abstract])) OR (Athletic[Title/Abstract])))) AND (((((((((Vision[Title/Abstract]) OR (Ocular Vision[Title/Abstract])) OR (Light Signal Transduction, Visual[Title/Abstract])) OR (Visual Light Signal Transduction[Title/Abstract])) OR (Visual Transduction[Title/Abstract])) OR (Transduction, Visual[Title/Abstract])) OR (Visual Phototransduction[Title/Abstract])) OR (Phototransduction, Visual[Title/Abstract])) OR (("Myopia"[Mesh]) OR ((Myopias[Title/Abstract]) OR (Nearsightedness[Title/Abstract]))))) AND (randomized controlled trial[Publication Type] OR randomized[Title/Abstract] OR placebo[Title/Abstract])

("Exercise"[MeSH Terms] OR ("physical activity"[Title/Abstract] OR "activities physical"[Title/Abstract] OR "activity physical"[Title/Abstract] OR "physical activities"[Title/Abstract] OR "exercise physical"[Title/Abstract] OR "exercises physical"[Title/Abstract] OR "physical exercise"[Title/Abstract] OR "physical exercises"[Title/Abstract] OR "acute exercise"[Title/Abstract] OR "acute exercises"[Title/Abstract] OR "exercise acute"[Title/Abstract] OR "exercises acute"[Title/Abstract] OR "exercise isometric"[Title/Abstract] OR "exercises isometric"[Title/Abstract] OR "isometric exercises"[Title/Abstract] OR "isometric exercise"[Title/Abstract] OR "exercise aerobic"[Title/Abstract] OR "aerobic exercise"[Title/Abstract] OR "aerobic exercises"[Title/Abstract] OR "exercises aerobic"[Title/Abstract] OR "exercise training"[Title/Abstract] OR "exercise trainings"[Title/Abstract] OR "training exercise"[Title/Abstract] OR (("education"[MeSH Subheading] OR "education"[All Fields] OR "Training"[All Fields] OR "education"[MeSH Terms] OR "train"[All Fields] OR "train s"[All Fields] OR "trained"[All Fields] OR "training s"[All Fields] OR "Trainings"[All Fields] OR "trains"[All Fields]) AND "Exercise"[Title/Abstract])) OR ("Sports"[MeSH Terms] OR ("Sport"[Title/Abstract] OR "Athletics"[Title/Abstract] OR "Athletic"[Title/Abstract]))) AND ("Vision"[Title/Abstract] OR "ocular vision"[Title/Abstract] OR ((("Light"[MeSH Terms] OR "Light"[All Fields] OR "lighted"[All Fields] OR "lights"[All Fields] OR "lighting"[MeSH Terms] OR "lighting"[All Fields] OR "lightings"[All Fields] OR "lightness"[All Fields] OR "lightnesses"[All Fields]) AND ("signal transduction"[MeSH Terms] OR ("Signal"[All Fields] AND "Transduction"[All Fields]) OR "signal transduction"[All Fields] OR "signaling"[All Fields] OR "Signal"[All Fields] OR "signal s"[All Fields] OR "signaled"[All Fields] OR "signaler"[All Fields] OR "signaler s"[All Fields] OR "signalers"[All Fields] OR "signalings"[All Fields] OR "signalization"[All Fields] OR "signalled"[All Fields] OR "signaller"[All Fields] OR "signaller s"[All Fields] OR "signallers"[All Fields] OR "signalling"[All Fields] OR "signallings"[All Fields] OR "signals"[All Fields])) AND "transduction visual"[Title/Abstract]) OR (("Visual"[All Fields] OR "visualisation"[All Fields] OR "visualisations"[All Fields] OR "visualise"[All Fields] OR "visualised"[All Fields] OR "visualises"[All Fields] OR "visualising"[All Fields] OR "visualization"[All Fields] OR "visualizations"[All Fields] OR "visualize"[All Fields] OR "visualized"[All Fields] OR "visualizer"[All Fields] OR "visualizers"[All Fields] OR "visualizes"[All Fields] OR "visualizing"[All Fields] OR "visually"[All Fields] OR "visuals"[All Fields]) AND "light signal transduction"[Title/Abstract]) OR "visual transduction"[Title/Abstract] OR "transduction visual"[Title/Abstract] OR "visual phototransduction"[Title/Abstract] OR "phototransduction visual"[Title/Abstract] OR ("Myopia"[MeSH Terms] OR ("Myopias"[Title/Abstract] OR "Nearsightedness"[Title/Abstract]))) AND ("randomized controlled trial"[Publication Type] OR "randomized"[Title/Abstract] OR "placebo"[Title/Abstract])

Translations

Trainings: "education"[Subheading] OR "education"[All Fields] OR "training"[All Fields] OR "education"[MeSH Terms] OR "train"[All Fields] OR "train's"[All Fields] OR "trained"[All Fields] OR "training's"[All Fields] OR "trainings"[All Fields] OR "trains"[All Fields]

Light: "light"[MeSH Terms] OR "light"[All Fields] OR "lighted"[All Fields] OR "lights"[All Fields] OR "lighting"[MeSH Terms] OR "lighting"[All Fields] OR "lightings"[All Fields] OR "lightness"[All Fields] OR "lightnesses"[All Fields]

Signal: "signal transduction"[MeSH Terms] OR ("signal"[All Fields] AND "transduction"[All Fields]) OR "signal transduction"[All Fields] OR "signaling"[All Fields] OR "signal"[All Fields] OR "signal's"[All Fields] OR "signaled"[All Fields] OR "signaler"[All Fields] OR "signaler's"[All Fields] OR "signalers"[All Fields] OR "signalings"[All Fields] OR "signalization"[All Fields] OR "signalled"[All Fields] OR "signaller"[All Fields] OR "signaller's"[All Fields] OR "signallers"[All Fields] OR "signalling"[All Fields] OR "signallings"[All Fields] OR "signals"[All Fields]

Visual: "visual"[All Fields] OR "visualisation"[All Fields] OR "visualisations"[All Fields] OR "visualise"[All Fields] OR "visualised"[All Fields] OR "visualises"[All Fields] OR "visualising"[All Fields] OR "visualization"[All Fields] OR "visualizations"[All Fields] OR "visualize"[All Fields] OR "visualized"[All Fields] OR "visualizer"[All Fields] OR "visualizers"[All Fields] OR "visualizes"[All Fields] OR "visualizing"[All Fields] OR "visually"[All Fields] OR "visuals"[All Fields]
