# Supplementary material for: Meta-analysis of the effects of physical activity on ocular biometrics in children and adolescents
Source: Front Public Health. 2025 Jun 11;13:1615033. doi: 10.3389/fpubh.2025.1615033 (PMC12187683; doi:10.3389/fpubh.2025.1615033)
Supplement: Supplementary file 1 [file Data_Sheet_1.ZIP › Search terms.docx]

# **Sport**

Athletics

Athletic

# **Exercise**

Exercises

Physical Activity

Activities, Physical

Activity, Physical

Physical Activities

Exercise, Physical

Exercises, Physical

Physical Exercise

Physical Exercises

Acute Exercise

Acute Exercises

Exercise, Acute

Exercises, Acute

Exercise, Isometric

Exercises, Isometric

Isometric Exercises

Isometric Exercise

Exercise, Aerobic

Aerobic Exercise

Aerobic Exercises

Exercises, Aerobic

Exercise Training

Exercise Trainings

Training, Exercise

Trainings, Exercise

# **Vision, Ocular**

Vision

Ocular Vision

Light Signal Transduction, Visual

Visual Light Signal Transduction

Visual Transduction

Transduction, Visual

Visual Phototransduction

Phototransduction, Visual

# **Myopia**

Myopias

Nearsightedness

Nearsightednesses
