# Supplementary material for: Meta-analysis of the effects of physical activity on ocular biometrics in children and adolescents
Source: Front Public Health. 2025 Jun 11;13:1615033. doi: 10.3389/fpubh.2025.1615033 (PMC12187683; doi:10.3389/fpubh.2025.1615033)
Supplement: Supplementary file 1 [file Data_Sheet_1.ZIP › Table1-4.docx]

Table 1 Descriptive characteristics of included studie

| Study | Country/region | Research desgin | Participants Characteristics | | | Intervention Measures | | | | Outcome Indicators |
| --- | --- | --- | --- | --- | --- | --- | --- | --- | --- | --- |
|  |  |  | Gender | Age range | Sample Size(T/C) | Intervention(T/C) | Cycle(week) | Frequency(Time/week) | Duration(minutes) |  |
| Sheng Zhou,2023 | China,Suzhou | RCT | NA | 10-11 | T=115,C=38 | Ciliary muscle training combined with physical education classes/Regular physical education classes | 32 | 3 | 40 | UDVA,KVA,AL |
| Pei-Chang Wu,2017 | China,Taiwan | RCT | M-52.15% | 6-7 | T=267,C=426 | 11 hours of outdoor activity per week, with an additional 150 minutes of exercise time each week/ROCT711 intervention was not conducted | 52 | NA | NA | The changes in SER and AL, as well as the variations in outdoor light intensity and duration |
| YIN,2024 | China,Suzhou | RCT | NA | 9-10 | T=43,C=43 | Physical activities (basketball/soccer) combined with ciliary muscle adjustment training/Regular physical activities | 16 | 3 | 40 | UDVA, KVA |
| Yin,2022 | China,Suzhou | RCT | M-59% | 10-11 | T=127,C=33 | Ciliary muscle training combined with open-skill exercises and closed-skill exercises/Regular physical activities | 16 | 3 | 40 | UDVA, KVA |
| Yingfeng Zheng,2021 | China,Guangdong | RCT | M-52.3% | 12-13 | T=467,C=429 | Home quarantine physical activities and REAP live broadcasts/Health information course (home exercise) | 2 | 7 | 15*4 | Eye strain |
| Mingguang He,2015 | China,Guangzhou | RCT | M-54% | 6-7 | T=902,C=913 | Outdoor activity course/Continue the previous activity mode | 156 | NA | NA | The changes in myopia rate, spherical SER, and AL |
| Ju-Xiang Jin,2015 | China,Shenyang | RCT | M-51% | 6-11 | T=214,C=177 | Outdoor activities (skipping rope and badminton)/No intervention measures | 52 | 5 | 40 | The incidence of new myopia RE,AL,IOP |
| Siegrist,2018 | Germany | RCT | M-60% | 10-11 | T=241,C=191 | Lifestyle course, with increased physical activities both on and off campus/JuvenTUM 3 | 78 | NA | NA | AVR,CRVE,CRAE |
| Guo,2019 | China,Beijing | nRCT | M-49% | 6-7 | T=157,C=216 | Jogging/No intervention measures | 52 | 5 | 30 | AL,RE |
| Pei-Chang Wu,2013 | China,Taiwan | nRCT | M-51% | 7-11 | T=333,C=238 | Outdoor activity (ROC)/Routine activities | 104 | NA | NA | SER,AL |

T Experimental group,C Control group,UDVA Uncorrected distance visual acuity,KVA Kinetic visual acuity,AL (mm) Axial length,SER Spherical Equivalent Refraction,RE Refractive error, IOP (mmHg) Intraocular pressure, AVR arteriolar-to-venular ratio,CRVE (μm) Central retinal venular equivalents, CRAE (μm) Central retinal arteriolar equivalents, NA Not applicable.

Table 2 Descriptive characteristics of included studie

| Study | Country/region | Research desgin | Participants Characteristics | | | Testing method | Outcome Indicators |
| --- | --- | --- | --- | --- | --- | --- | --- |
|  |  |  | Gender | Age range/mean±sd | Sample Size |  |  |
| Nina Jacobsen,2008 | Denmark | Prospective cohort study | M-39% | 23.1±3.3 | Total=143 | Power bike | SER,AL |
| Kathryn A. Rose,2008 | Australia | Cross-sectional study | NA | 6 and 12 | 6year=1735 | NA | SER |
|  |  |  |  |  | 12year=2353 |  |  |
| Bamini Gopinath,2024 | Australia | Cross-sectional study | M-50.7% | 6-7 | Total=1492 | NA | CRAE、CRVE |
| K. Imhof,2016 | Switzerland | Cross-sectional study | M-53% | 7.3±0.4 | Total=1255 | 20-m Shuttle run | AVR,CRAE,CRVE |

AL (mm) Axial length,SER Spherical Equivalent Refraction, AVR arteriolar-to-venular ratio,CRVE (μm) Central retinal venular equivalents, CRAE (μm) Central retinal arteriolar equivalents, NA Not applicable.

Table 3 Summary of methodological quality of the included studies according to the PEDro scale (n = 11).

| Study | Eligibility criteria | Random allocation | Concealed allocation | Baseline comparability | Blinded subjects | Blinded therapist | Blinded assessor | Sufficient follow-up sample size | Intention-to-treat analysis | Intergroup comparison | Estimation and variability | Total score/11 |
| --- | --- | --- | --- | --- | --- | --- | --- | --- | --- | --- | --- | --- |
| ShengZhou,2023 | Y | Y | N | Y | N | N | N | Y | Y | Y | Y | 7 |
| Pei-Chang Wu,2017 | Y | Y | Y | Y | N | N | Y | Y | Y | Y | Y | 9 |
| YIN,2024 | Y | Y | N | Y | N | N | N | Y | Y | Y | Y | 7 |
| YIN,2022 | Y | Y | N | Y | N | N | N | Y | Y | Y | Y | 7 |
| YIingfeng Zheng,2021 | Y | Y | Y | Y | N | N | Y | Y | Y | Y | Y | 9 |
| Mingguang He,2015 | Y | Y | Y | Y | N | N | N | Y | Y | Y | Y | 8 |
| Ju-Xiang Jin,2015 | Y | Y | N | Y | N | N | N | Y | Y | Y | Y | 7 |
| Siegrist,2018 | Y | Y | Y | Y | N | N | N | Y | Y | Y | Y | 8 |
| Guo.2019 | Y | N | N | Y | N | N | N | Y | Y | Y | Y | 6 |
| Pei-Chang Wu,2013 | Y | N | N | Y | N | N | N | Y | Y | Y | Y | 6 |

Each item is scored as 1 point(Y) for compliance and 0 points(N) for non-compliance; the highest score is 9 points (it is not possible to blind subjects and therapists in intervention studies

Table 4 The methodological quality of cohort studies and cross-sectional studies included in the research is summarized according to the NOS (Newcastle-Ottawa Scale) assessment scale (n=4)

| Study | Selection | | | | Outcom | | | Comparability | Total score(9) |
| --- | --- | --- | --- | --- | --- | --- | --- | --- | --- |
|  | Representativeness of the exposed cohort(★) | Selection of the non exposed cohort(★) | Ascertainment of exposure(★) | Demonstration that outcome of interest was not present at start of study(★) | Assessment of outcome(★) | Was follow-up long enough for outcomes to occur(★) | Adequacy of follow up of cohorts(★) | Comparability of cohorts on the basis of the design or analysis(2★) |  |
| Kathryn A. Rose,2008 | ★ | ★ | ★ |  | ★ | ★ | ★ | ★★ | 8 |
| Bamini Gopinath,2011 | ★ | ★ | ★ | ★ | ★ | ★ | ★ | ★★ | 9 |
| K. Imhof,2016 |  | ★ | ★ | ★ | ★ |  |  | ★★ | 6 |
| Nina Jacobsen,2008 | ★ | ★ | ★ | ★ | ★ | ★ | ★ | ★★ | 9 |

In the two main categories of selection and outcomes, each subcategory can earn a maximum of 1★, with comparability earning a maximum of 2★. The highest possible score is 9★
