# Supplementary figures and images for: Meta-analysis of the effects of physical activity on ocular biometrics in children and adolescents
Source: Front Public Health. 2025 Jun 11;13:1615033. doi: 10.3389/fpubh.2025.1615033 (PMC12187683; doi:10.3389/fpubh.2025.1615033)

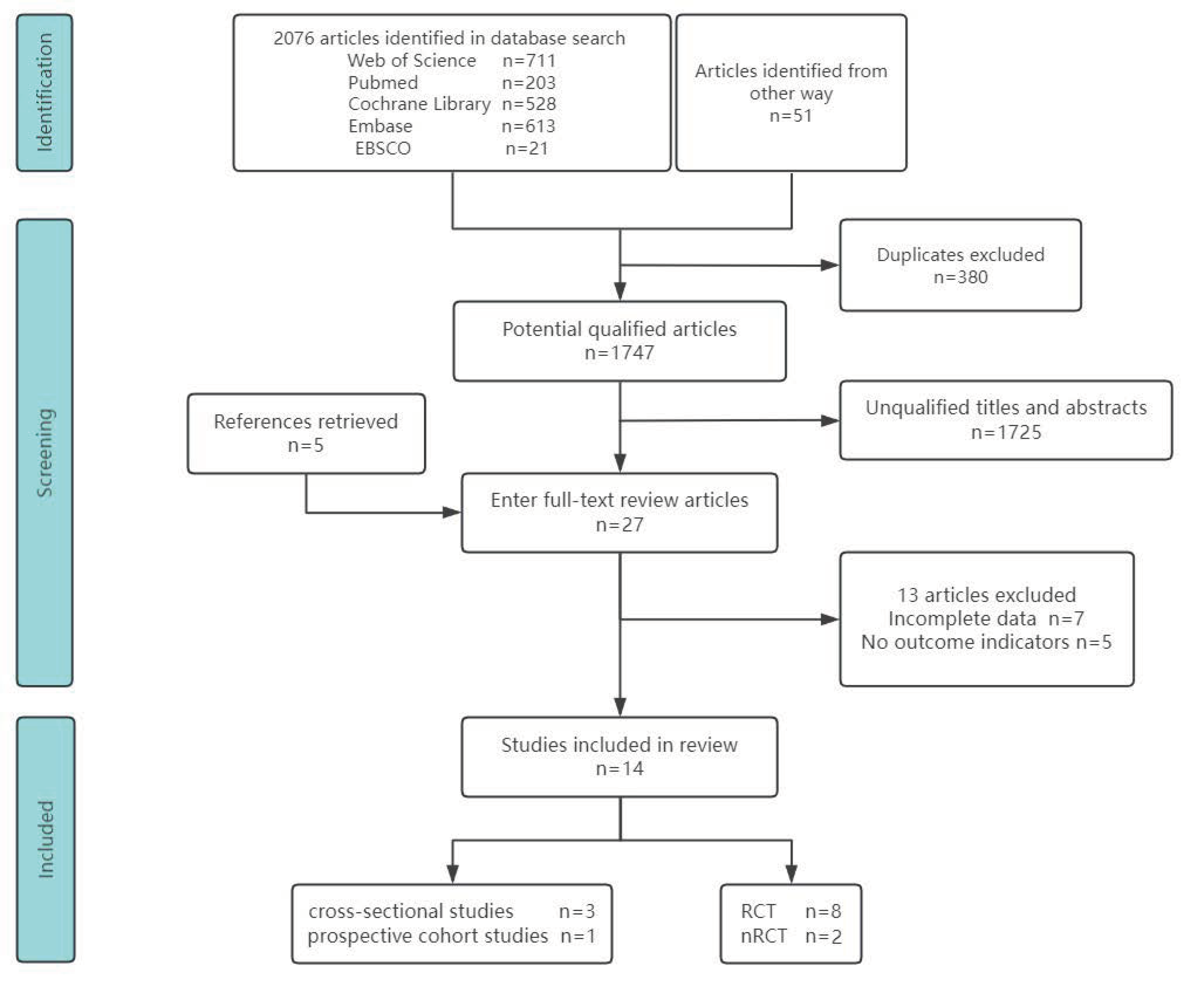

Supplement: Supplementary file 1 [file Data_Sheet_1.ZIP › Figure 1.tif]

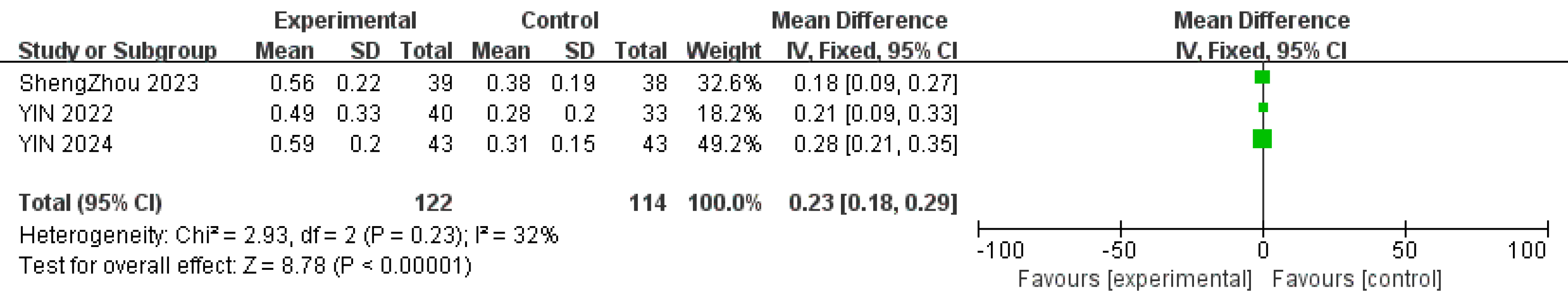

Supplement: Supplementary file 1 [file Data_Sheet_1.ZIP › Figure 2.tif]

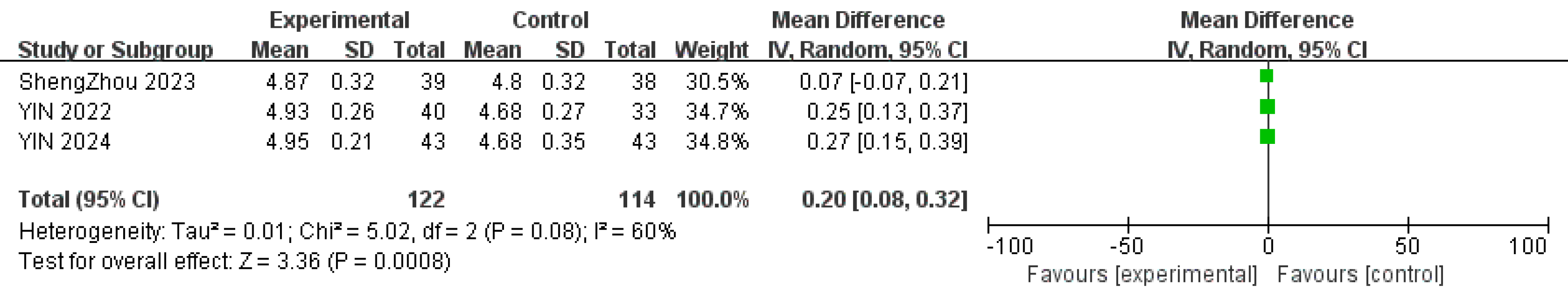

Supplement: Supplementary file 1 [file Data_Sheet_1.ZIP › Figure 3.tif]

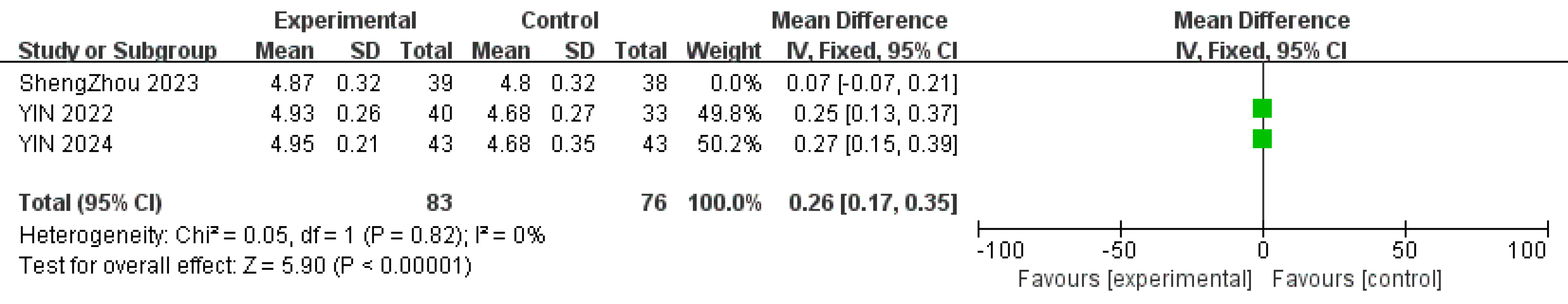

Supplement: Supplementary file 1 [file Data_Sheet_1.ZIP › Figure 4.tif]

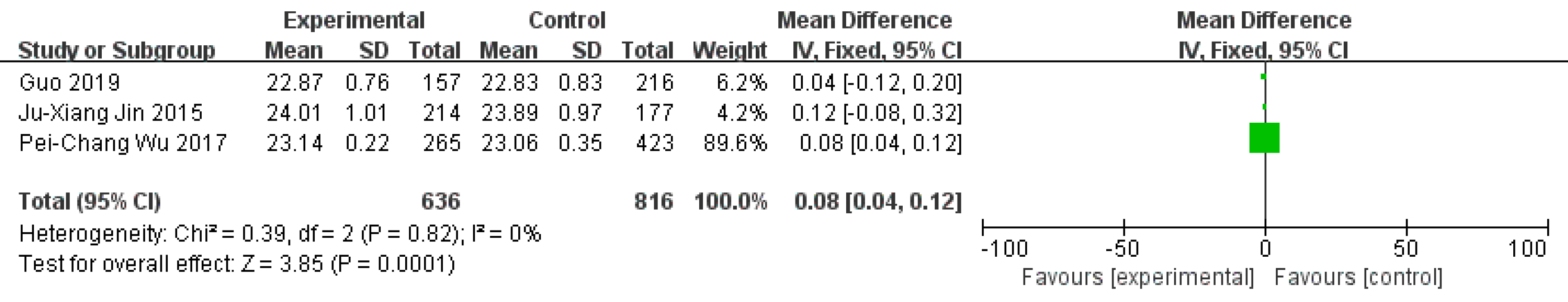

Supplement: Supplementary file 1 [file Data_Sheet_1.ZIP › Figure 5.tif]

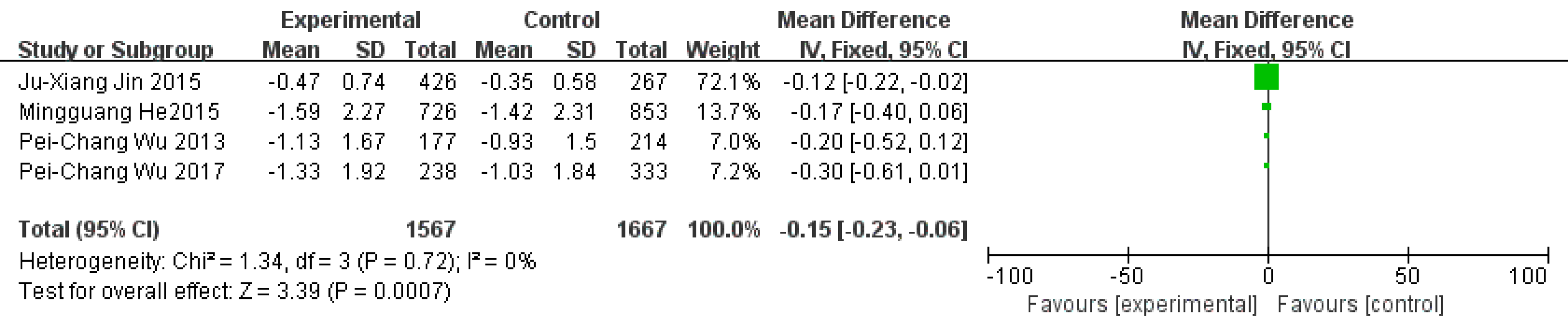

Supplement: Supplementary file 1 [file Data_Sheet_1.ZIP › Figure 6.tif]

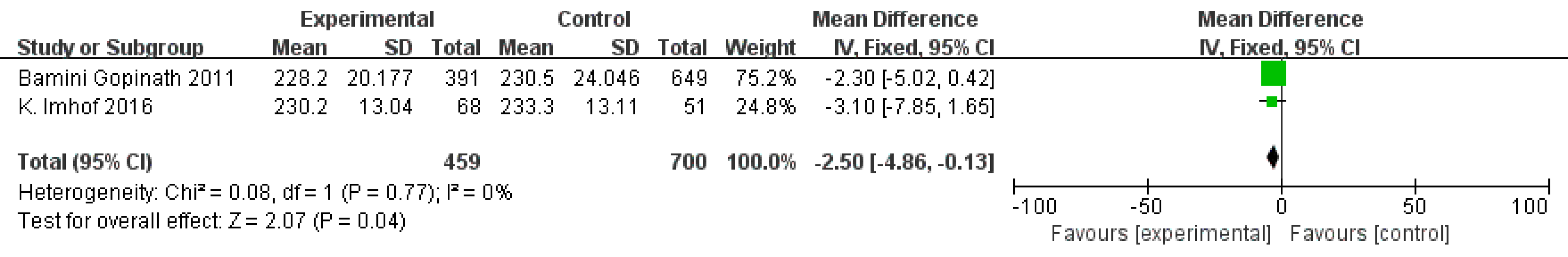

Supplement: Supplementary file 1 [file Data_Sheet_1.ZIP › Figure 7.tif]

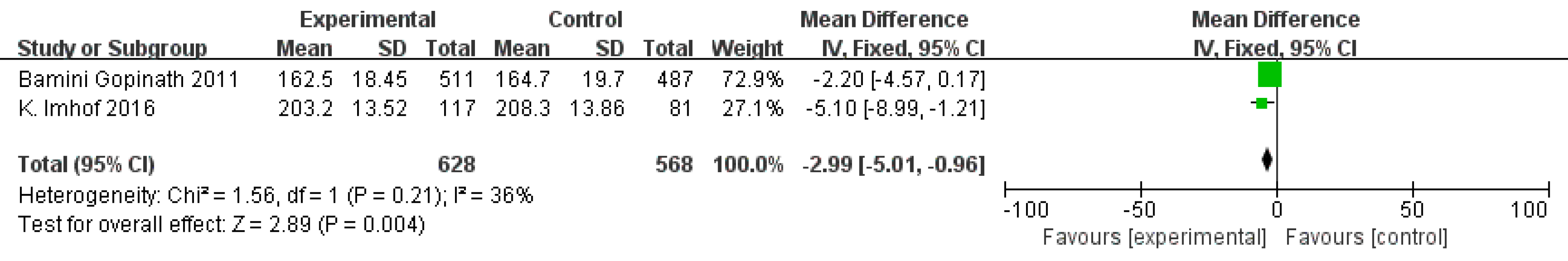

Supplement: Supplementary file 1 [file Data_Sheet_1.ZIP › Figure 8.tif]
